# Brain white matter microstructural changes in chemotherapy‐treated older long‐term breast cancer survivors

**DOI:** 10.1002/cam4.6881

**Published:** 2023-12-28

**Authors:** Ebenezer Daniel, Frank Deng, Sunita K. Patel, Mina S. Sedrak, Heeyoung Kim, Marianne Razavi, Can‐Lan Sun, James C. Root, Tim A. Ahles, William Dale, Bihong T. Chen

**Affiliations:** ^1^ Department of Diagnostic Radiology City of Hope National Medical Center Duarte CA USA; ^2^ Department of Population Science City of Hope National Medical Center Duarte CA USA; ^3^ Department of Medical Oncology City of Hope National Medical Center Duarte CA USA; ^4^ Center for Cancer and Aging City of Hope National Medical Center Duarte CA USA; ^5^ Department of Supportive Care Medicine City of Hope National Medical Center Duarte CA USA; ^6^ Neurocognitive Research Lab Memorial Sloan Kettering Cancer Center New York NY USA

**Keywords:** breast cancer, cancer‐related cognitive impairment, chemotherapy, diffusion tensor imaging, tract‐based spatial statistics

## Abstract

**Purpose:**

To assess white matter microstructural changes in older long‐term breast cancer survivors 5–15 years post‐chemotherapy treatment.

**Methods:**

Breast cancer survivors aged 65 years or older who underwent chemotherapy (C+) and who did not undergo chemotherapy (C−) and age‐ and sex‐matched healthy controls (HC) were enrolled at time point 1 (TP1) and followed for 2 years for time point 2 (TP2). All participants underwent brain MRI with diffusion tensor images and neuropsychological (NP) testing with the NIH Toolbox Cognition Battery. Tract‐based spatial statistics (TBSS) analysis was performed on the diffusion tensor images to assess white matter microstructural changes with the fractional anisotropy (FA) parameter.

**Results:**

There were significant longitudinal alterations in FA within the C+ group over time. The C+ group showed diminished FA in the body and genu of corpus callosum, anterior corona radiate, and external capsule on both the whole brain and region of interest (ROI) based analyses after *p* < 0.05 family‐wise error (FWE) correction. However, there were no significant group differences between the groups at TP1. Additionally, at TP1, a positive correlation (*R* = 0.58, *p* = 0.04) was observed between the FA value of the anterior corona radiata and the crystallized composite score in the C+ group.

**Conclusions:**

Brain white matter microstructural alterations may be the underlying neural correlates of cognitive changes in older breast cancer survivors who had chemotherapy treatment years ago.

## 1 INTRODUCTION

Cancer‐related cognitive impairment (CRCI) is an increasing issue in cancer survivors, especially in older survivors.^1^ Recent advancements in cancer treatment, such as targeted therapies, immunotherapy, and endocrine therapy, have improved breast cancer survival.^2^, ^3^ CRCI has been reported in cancer survivors, which affects their quality of life.^4^ Older survivors may have persistent CRCI, particularly in attention, processing speed, working memory, and executive function, even several years after treatment.^5^, ^6^ In addition, older cancer survivors treated with chemotherapy may be more susceptible to cognitive impairment than younger cohorts and cancer control groups.^7^ However, there are few studies focusing on the long‐term effects of chemotherapy on older cancer survivors.

Neuroimaging has been used to assess the neural correlates of CRCI in cancer survivors treated with chemotherapy.^8^ Brain white matter imaging, such as diffusion tensor imaging (DTI), could be used to evaluate white matter microstructural integrity in patients with CRCI.^9^ Significantly diminished white matter microstructural integrity has been reported in the superior longitudinal fasciculus and corticospinal tract of breast cancer patients 6 months after chemotherapy treatment.^10^ Our own study of older breast cancer patients showed diminished fractional anisotropy (FA) in the genu of the corpus callosum shortly after chemotherapy.^11^ An older longitudinal study showed white matter changes in frontal, parietal, and occipital regions from pretreatment to 4 months post‐chemotherapy,^12^ and the same research group demonstrated recovery in a follow‐up study at 3–4 years after chemotherapy in breast cancer survivors.^13^ Another research group showed decreased FA in the cerebral peduncle, inferior fronto‐occipital fasciculus, thalamic radiation, and longitudinal fasciculi 3 years after chemotherapy.^14^ In addition, chemotherapy‐treated breast cancer survivors have shown long‐term white matter changes even 20 years after treatment.^15^ Overall, the literature has shown white matter changes acutely after chemotherapy, with some evidence of both recovery and persistent alterations over time. The previous studies were mostly based on relatively younger patients, while the long‐term impacts of chemotherapy on the white matter microstructure of breast cancer survivors in older survivors are largely unknown.

In this prospective longitudinal study, we enrolled older long‐term breast cancer survivors with or without chemotherapy and an age‐ and sex‐matched healthy control group (HC group) followed over 2 years. We aimed to investigate the long‐term changes in white matter microstructure in older, long‐term breast cancer survivors 5–15 years after chemotherapy treatment. We analyzed brain DTI data to assess white matter changes with FA values and used neuropsychological (NP) testing scores to assess cognitive performance. We hypothesized that the chemotherapy‐treated group would have diminished FA values that would be correlated with the NP scores.

## 2 METHODS

### 2.1 Participants

We enrolled older breast cancer survivors aged ≥65 years treated with chemotherapy (C+) 5–15 years prior to enrollment, age‐, and sex‐matched breast cancer controls without chemotherapy (C−), and healthy controls (HC) at time point 1 (TP1). The participants were followed for up to 2 years (TP2). Subjects were excluded for a history of psychiatric disease, other cancer diagnosis or treatment, neurodegenerative disorder, metastatic disease, or stroke. All were right‐handed participants. In the HC group, we excluded participants with any history of cancer, in addition to the abovementioned criteria for cancer survivors. However, the HC group was included in the study without any prior assessment of cognitive impairment or white matter‐related changes. The HC subjects had no history of cancer and were recruited through patients' referrals, local newspaper advertisements, and community health fairs. This neuroimaging study was a substudy of the parent trial, Cognition in older breast cancer survivors: treatment exposure, APOE, and smoking history (NCT02122107), and written informed consent was obtained from all participants. This study was approved by the institutional review board (IRB) of the City of Hope National Medical Center.

### 2.2 Cognitive testing

The NIH Toolbox Cognition Battery^16^, ^17^ was used for neuropsychological testing. This cognitive testing battery generated seven scores that measured different cognitive abilities, including picture sequence memory, pattern comparison processing speed, picture vocabulary, oral reading recognition, list sorting working memory, flanker inhibitory control, and dimensional change card sorting. Composite scores for crystallized, fluid, and total composite function were also obtained from the cognition battery.

A generalized linear model (GLM) was utilized to account for repeated measurements within subjects. The GLM analyzed group (C+; C−; HC) and time point (TP1; TP2) as categorical fixed effects. Three tests were performed using the GLM: (1) testing the difference in the NP scores between the groups at TP1, (2) identifying significant longitudinal differences within the groups, and (3) testing the group by time interaction effect. Data analysis was performed using SAS 9.3 (SAS Institute). The detailed results of the NP scores for this cohort have been previously published in our cortical thickness study.^18^

### 2.3 DTI data acquisition and processing

The same Verio 3 T scanner (Siemens, Erlangen, Germany) was used for all brain MRI scans. The DTI data was obtained with the following parameters: 20 directions, repetition time (TR) of 9400 milliseconds (ms), echo time (TE) of 87 ms, field of view (FOV) of 224 × 224 mm^2^, flip angle = 90°, voxel size of 1.75 × 1.75 × 2.34 mm^3^, 64 slices, and a *p*‐value of 1000 s/mm^2^. All brain MRI scans were evaluated for incidental brain abnormalities by the study neuroradiologist (BTC).

The FMRIB Software Library (FSL) version 6.0 at http://www.fmrib.ox.ac.uk/fsl was utilized to perform tract‐based spatial statistics (TBSS) analysis.^19^, ^20^ Before processing, data was evaluated for significant artifacts or apparent data loss, and if detected, such data were eliminated. Subsequently, brain masks were generated using the brain extraction tool (BET).^21^ To generate a series of three eigenvectors and three eigenvalues, FMRIB's Diffusion Toolbox (FDT) fitting of the diffusion tensor was performed on each voxel present in the brain using the FSL software. These eigenvalues were then utilized to calculate the FA diffusion metrics, as in our prior study on a different cohort.^11^

### 2.4 Whole brain and region of interest (ROI) analyses

To perform the whole brain FA white matter analysis using TBSS, the FA images were preprocessed and registered to the Montreal Neurological Institute (MNI) standard space with a resolution of 1 × 1 × 1 mm^3. To generate the mean FA skeleton image, the FA images from each group were combined to form a merged image, referred to as the “all FA” image. From the combined image, the average FA value was computed and utilized to produce the image of the “mean FA skeleton.” Afterwards, the skeleton was subjected to a default threshold of 0.3. A GLM was created using a graphical user interface (GUI) tool in the FSL software to assess the statistical changes in the FA skeleton. The “two sample unpaired *t*‐test” was used to test the difference in the whole brain FA skeleton between the groups. Meanwhile, the changes within groups from TP1 to TP2 were evaluated using the “paired *t*‐test.” The interaction between the group and time was assessed using the “two‐way between‐subjects” ANOVA’ and “three‐way between‐subjects' ANOVA” within the FSL GUI model. After correcting for family‐wise error (FWE) using a threshold of *p* < 0.05, the statistical significance was evaluated at the cluster level.

To perform the ROI analysis, we used the JHU white matter template to extract the mean FA values from specific white matter tracts for all participants in the study. The ROI was identified as the white matter tracts showing significant longitudinal changes in the C+ group during the whole‐brain analysis. The tracts that were measured included the left anterior corona radiata, body of the corpus callosum, genu of the corpus callosum, and left external capsule. To evaluate the statistical significance of the ROI‐based data at TP1, an “independent sample *t*‐test” was used to compare the groups. Subsequently, “paired *t*‐tests” were conducted to assess within the group differences overtime, and group by time interaction was evaluated using the “linear mixed model” analysis. The IBM statistical package for social sciences (SPSS v.27) was used to test the differences in the FA values from the ROI analysis. A *p* value <0.05 was deemed to be significant, although formal comparisons were only exploratory.

### 2.5 Correlation analysis

To evaluate the correlation between the mean ROI values of the left anterior corona radiata, body of corpus callosum, genu of corpus callosum, and left external capsule and the three composite scores, including the crystallized, fluid, and total composite scores, linear regression analyses were performed. The correlation analysis was done at TP1. In addition, correlations were also performed between the FA changes and the composite score changes. A *p* value at a threshold less than 0.05 was considered statistically significant.

## 3 RESULTS

### 3.1 Clinical and demographic data

This study cohort consisted of 60 participants at TP1, among which 20 were in the C+, C−, and HC groups, respectively. There was severe attrition of this study cohort due to various reasons, including patients with new memory problems, new cancer, refusal to continue with the study, loss of follow‐up, and death. The final cohort with both TP1 and TP2 data included 12 subjects for each of the three groups. Attrition at TP2 for the longitudinal evaluation occurred across the study groups. In the C+ group, attrition occurred due to multiple factors: three participants developed new cancer, one confronted memory problems, two refused to continue, one relocated, and one participant passed away. Similarly, in the C− group, attrition involved five subjects who were lost to follow‐up, two who refused to continue, and one who relocated. The HC group also faced attrition, with three participants lost to follow‐up and two who moved away during the study.

As shown in Table 1, there were no significant differences in age (*p* = 0.75), education (*p* = 0.80), or race (*p* = 0.37) among the three groups. Additional clinical and demographic information about the cohort has been reported in our cortical thickness study.^18^ The chemotherapy regimens used in our C+ group were distributed as follows: 9 (75%) participants received TC (docetaxel and cyclophosphamide), 1 (8%) participant received AC (doxorubicin and cyclophosphamide), 1 (8%) participant received CMF (cyclophosphamide, methotrexate, and 5‐fluorouracil), and 1 (8%) participant received TAC (Taxotere, Adriamycin, and cyclophosphamide).

**TABLE 1:** Demographic and clinical information.

Parameters	C+*, N* = 12	C−*, N* = 12	HC*, N* = 15	*p*
Age (years)				
Mean (SD)	73.75 (5.41)	76.50 (4.28)	74.53 (6.73)	0.48
Median (Range)	71.50 (68–84)	75.5 (71–86)	73.00 (66–88)	
Race (*N*, %)				
White or Caucasian	10 (83)	11 (92)	14 (93)	0.76
Black	1 (8)	1 (8)	–	
Other	1 (8)	–	1 (7)	
Ethnicity (*N*, %)				
Not Hispanic	10 (83)	12 (100)	13 (87)	0.53
Hispanic	2 (17)	–	2 (13)	
Education (*N*, %)				
High school or less	3 (25)	4 (33)	4 (27)	0.99
College or above	9 (75)	8 (67)	11 (73)	
AJCC Stage (*N*, %)				
DCIS	1 (8)	6 (50)		
I	1 (8)	4 (33)		
II	10 (83)	2 (17)		
III				
Chemotherapy (Regimen) (*N*, %)				
TC	9 (75)			
AC	1 (8)			
CMF	1 (8)			
TAC	1 (8)			

*Note*: In the C+ group, years from diagnosis to TP1 (Mean, SD): 7.42 (2.34). In the C− group, years from diagnosis to TP1 (Mean, SD): 8.20 (2.86). Based on the parameter years from diagnosis (to TP1), there was no mean difference between the C+ and the C− group (*p* = 0.372). In the C+ group, the year ending of chemotherapy to TP1 (Mean, SD) is 6.52 (2.33). The parameters were statistically significant when the *p* value was less than 0.05.

Abbreviations: AC, doxorubicin (Adriamycin) and cyclophosphamide; AJCC, American Joint Committee on Cancer; C−, no‐chemotherapy group; C+, chemotherapy group; CMF, cyclophosphamide, methotrexate, and 5 fluorouracil (5 FU); DCIS, Ductal Carcinoma in Situ. ANOVA or Fisher tests were utilized, depending on whether the data was continuous or categorical; HC, healthy control group; N, number of subjects; SD, standard deviation; TAC, (Taxotere, Adriamycin, and cyclophosphamide); TC, docetaxel and cyclophosphamide.

### 3.2 FA data from whole brain analysis

**FIGURE 1:**
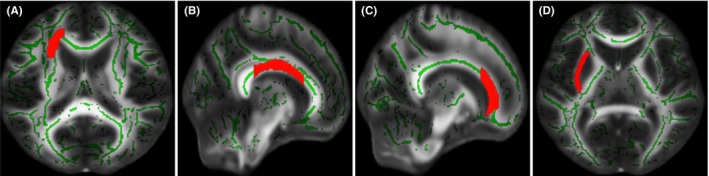
White matter tracts showing significantly decreased fractional anisotropy values from time point 1 to time point 2 in the chemotherapy‐treated group (red highlighted regions). A. Left anterior corona radiata. B. Body of corpus callosum. C. Genu of corpus callosum. D. Left external capsule.

The voxel‐wise whole‐brain TBSS analysis found no significant differences in the FA values between the groups (C+ vs. C−, C+ vs. HC, and C− vs. HC) at TP1. The longitudinal analysis for within‐group differences showed decreased FA values in the left anterior corona radiata, body of corpus callosum, genu of corpus callosum, and left external capsule in the C+ group from whole brain analysis (Figure 1 and Table 2). Decreased FA values were observed in the right superior corona radiata, right posterior thalamic radiation (including optic radiation), splenium of corpus callosum, right posterior corona radiata, and genu of corpus callosum in the C− group from whole brain analysis (Table 3). There were no significant differences observed within the HC group over time. These results remained significant after at a threshold of 0.05 based on FWE correction. No significant differences were observed based on group by time interaction among the three groups. Also, we assessed the possible association between the duration since chemotherapy and the years since cancer diagnosis with changes in white matter tracts. Our analysis revealed no significant associations between white matter tracts and these two variables, applying a significance threshold of 0.05. Detailed information about this analysis can be found in the supplementary materials, specifically in Tables S1 and S2.

**TABLE 2:** Within‐group changes from region of interest (ROI) analysis of white matter fractional anisotropy (FA) values from time point 1 (TP1) to time point 2 (TP2) for the three groups of study participants.

White matter tracts	MNI (x, y, z)	Mean (SD)			*t* value	*p* value	
		TP1	TP2	Changes		Paired *t*‐test	Wilcoxon signed ranks test
Anterior corona radiata left	(115, 144, 82)						
C+		0.45 (0.05)	0.43 (0.05)	0.02 (0.03)	2.60	**0.02**	**0.01**
C−		0.44 (0.03)	0.43 (0.03)	0.01 (0.02)	2.00	0.07	0.08
HC		0.44 (0.02)	0.43 (0.03)	0.01 (0.02)	1.71	0.10	0.06
Body of corpus callosum	(101,139, 98)						
C+		0.62 (0.05)	0.59 (0.06)	0.02 (0.03)	2.30	**0.04**	**0.02**
C−		0.62 (0.03)	0.60 (0.04)	0.01 (0.03)	1.70	0.11	0.14
HC		0.60 (0.04)	0.60 (0.06)	0.01 (0.04)	0.48	0.63	0.65
Genu of corpus callosum	(102, 156, 70)						
C+		0.51 (0.03)	0.48 (0.04)	0.03 (0.03)	3.30	**<0.01**	**<0.01**
C−		0.51 (0.02)	0.49 (0.03)	0.01 (0.01)	3.52	**<0.01**	**0.01**
HC		0.48 (0.05)	0.48 (0.05)	0.01 (0.03)	0.64	0.53	0.32
External capsule left	(117, 143, 74)						
C+		0.40 (0.02)	0.38 (0.03)	0.02 (0.02)	2.45	**0.03**	**0.02**
C−		0.51 (0.02)	0.49 (0.03)	0.01 (0.01)	3.52	**<0.01**	0.29
HC		0.36 (0.03)	0.36 (0.04)	0.01 (0.02)	1.06	0.30	0.33

*Note*: Significant *p* values set at <0.05.

Abbreviations: C+, chemotherapy group; C−, no‐chemotherapy group; HC, healthy control group; MNI, Montreal Neurological Institute; SD, standard deviation.

**TABLE 3:** White matter tracts showing significantly decreased fractional anisotropy (FA) values from time point 1 to time point 2 in the no‐chemotherapy (C−) group.

Region	Voxel location (MNI)	*p* value (voxel)
Superior corona radiata right	67, 107, 107	0.01
Posterior thalamic radiation (include optic radiation) right	57, 63, 79	0.03
Splenium of corpus callosum right	72, 79, 81	0.03
Posterior corona radiata right	67, 81, 101	0.01
Genu of corpus callosum	104, 156, 69	0.02

*Note*: Significant *p* value set at <0.05.

Abbreviation: MNI, Montreal Neurological Institute.

### 3.3 FA data from ROI analysis

We extracted the FA values from all subjects using an ROI approach. The ROI regions were the four regions with significantly decreased FA values in the whole brain analysis in the C+ group, which included the body and genu of corpus callosum, left anterior corona radiata, and left external capsule (Table 2). Table 2 presents the within‐group changes from the region of interest (ROI) analysis of white matter fractional anisotropy (FA) values from TP1 to TP2 for the three groups of study participants. Regarding the within‐group analysis over time, there was a significant decrease of FA values in the left anterior corona radiata in the C+ group (*p* = 0.02), whereas no significant decrease was noted in the C− (*p* = 0.07) or the HC (*p* = 0.10) group. The C+ group showed decreased FA values in the body of corpus callosum (*p* = 0.04), whereas there were no significant changes in FA values in the C− (*p* = 0.11) or the HC (*p* = 0.63) group. Both the C+ (*p* < 0.01) and C− (*p* < 0.01) groups showed significant FA decreases in the genu of corpus callosum, but the HC group showed no changes in FA values (*p* = 0.53). Finally, significant changes in FA values were noted in the left external capsule within the C+ group (*p* = 0.03) and C− (*p* < 0.01) groups overtime but not in the HC group (*p* = 0.30). No significant group by time interactions were noted.

Additionally, together with the parametric “paired *t*‐test,” we conducted the non‐parametric “Wilcoxon signed ranks test,” which produced comparable significant results within the C+ group for the left anterior corona radiata (*p* = 0.01), the body of the corpus callosum (*p* = 0.02), the genu of the corpus callosum (*p* < 0.01), and the left external capsule (*p* = 0.02). Though in the C‐ group, only the genu of the corpus callosum (*p* = 0.01) showed a significant reduction in the non‐parametric test.

### 3.4 Cognitive testing data

At TP1, the crystallized composite score (*p* = 0.04) and oral reading recognition score (*p* = 0.02) were significantly lower in the C+ group as compared to the C− group. Within the C+ group analysis, the total composite score (*p* = 0.01), fluid composite score (*p* = 0.03), and picture vocabulary scores decreased over time (*p* = 0.04). Additionally, the decreases in the crystalized composite score (*p* = 0.057) and picture sequence memory scores (*p* = 0.05) approached significance in the C+ group. There was no significant difference in the cognitive testing scores within the control groups. There were no significant group by time differences in the cognitive testing scores. The summary of NP testing scores is given in the supplementary file, Table S3. Also, the detailed NP scores were reported in our prior study focusing on cortical thickness analysis.^18^

### 3.5 Correlation results

**FIGURE 2:**
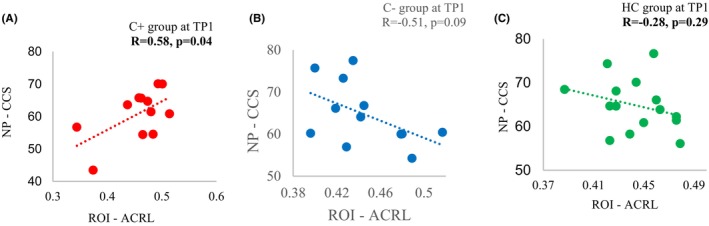
Correlation between the fractional anisotropy values in the left anterior corona radiata and the crystallized composite scores at time point 1 (TP1). (A) Chemotherapy (C+) group. (B) No‐chemotherapy (C−) group. (C) Healthy control (HC) group. *R*: the Pearson's correlation coefficient, with significance set at *p* ≤ 0.05. NP‐CCS, neuropsychological score—crystalized composite score; ROI—ACRL, region of interest—anterior corona radiata left.

There was a significant positive correlation between the crystallized composite score and the FA value in the left anterior corona radiata at TP1 (*p* = 0.04, *R* = 0.58) in the C+ group. There were no significant correlations observed in the C− or HC group (Figure 2).

## 4 DISCUSSION

In this study, we observed decreased FA of white matter in older long‐term survivors of breast cancer who had chemotherapy treatment 5–15 years prior. We also found a significant positive association between FA values and cognitive testing scores at TP1. To the best of our knowledge, this was the first prospective exploratory longitudinal study focusing on the impact of chemotherapy on brain white matter microstructural integrity in older, long‐term survivors of breast cancer.

We found decreased FA values in the body and genu of the corpus callosum in the chemotherapy‐treated group, which was consistent with the literature in younger survivors.^12^ A prior study of patients with breast cancer also showed decreased FA in the corpus callosum of patients treated with chemotherapy.^12^ Alterations in the corpus callosum may affect memory, visuospatial, and calculating abilities.^22^, ^23^ Changes in the body of the corpus callosum may affect the white matter pathway in the temporal, parietal, and occipital regions of the brain.^24^ In addition, damage to the body of corpus callosum has been reported in patients with Alzheimer's disease (AD) and in patients with mild cognitive impairment.^25^, ^26^ In this study, we found diminished white matter in the corpus callosum similar to what has been reported in AD studies, contributing novel information on the long‐term effects of chemotherapy on brain white matter integrity in older breast cancer survivors. In addition, FA reduction in the white matter tracts of the left external capsule could potentially contribute to executive dysfunction, as reported in the literature.^27^ Prior studies have identified degradation in the white matter bundles of the left external capsule as a characteristic biomarker in the aging population.^27^, ^28^ However, our study showed diminished FA in the left external capsule in the C+ group only and not in the control groups. We speculate this may be partly related to persistent neurotoxicity from the chemotherapy treatment in the older breast cancer patients.

Prior studies have shown the genu of corpus callosum to have a more pronounced negative correlation with aging than other parts of the corpus callosum.^22^, ^29^ Diminished white matter in the genu of the corpus callosum could affect the interconnection between the left and right dorsolateral prefrontal cortex, which is important for working memory functions.^30^ We found significantly decreased FA values in the genu of the corpus callosum in both groups of cancer survivors (C+ and C− groups) but not in the HC group. We also observed no significant differences in the decreased FA values between the two cancer survivor groups. Interestingly, the parent study from which the present neuroimaging substudy derived showed worse performance on all three domains of cognitive function, including language, executive function, and learning and memory, over time in the cancer survivors with or without chemotherapy but not in the non‐cancer controls. Similar to the FA data, the parent study found no significant differences in cognitive testing scores between the two cancer survivor groups.^31^ Taken together, the FA values in the genu of the corpus callosum and the cognitive functioning of the three domains could potentially serve as markers for CRCI in older breast cancer survivors.

We also found diminished FA in the corona radiata in our chemotherapy‐treated older long‐term survivors. The damage to the microstructural integrity of the corona radiata has been linked to cognitive impairment, particularly affecting working memory and processing speed in an aging population.^32^ Prior study of patients with breast cancer and chemotherapy showed the decreased FA in corona radiata being associated with attention.^33^ Overall, our study results support the speculation of prior studies, that is, the decline in FA could indicate demyelination and axonal injury in white matter tracts associated with chemotherapy effects.^12^, ^13^ The observed deficits in white matter tracts in our C+ group predominantly affected the left hemisphere. Our previous study showed a similar pattern of predilection for the left hemisphere with diminished cortical thickness in the chemotherapy‐treated group.^18^ Additionally, a study on AD patients reported a higher rate of gray matter decrease in the left hemisphere as compared to the right hemisphere.^34^ Notably, all the participants in our study were right‐handed, indicating a dominant left hemisphere in this cohort.^35^, ^36^ Nevertheless, the underlying mechanism for the FA changes in the left hemisphere in the C+ group is not clear, and more studies are needed to assess the hemispheric predilection.

Our study findings in the long‐term survivors were different from the results of previous studies focusing on the more acute effects of chemotherapy shortly after treatment.^12^, ^13^ The prior studies focusing on acute effects showed more extensive white matter alterations in corpus callosum and corona radiata than the current study.^12^, ^13^ Our own prior study on the acute effects of chemotherapy showed white matter deficits in the genu and splenium of corpus callosum,^11^ whereas the present study on the long‐term effects of chemotherapy showed decreased FA in the genu but not in the splenium of corpus callosum. We speculate that there might be a partial recovery of white matter after chemotherapy. This speculation was supported by a previous study that found diminished white matter microstructure in the corona radiata and corpus callosum during the first 3–5 months following chemotherapy,^12^ with subsequent recovery at 3–4 years after treatment.^13^ One potential explanation for the recovery might be the remyelination of previously damaged white matter.^12^, ^13^ It is reasonable to assume that the initial recovery may not be adequate nor sustainable, as the detrimental effect of chemotherapy on white matter may persist up to 9–10 years after treatment^37^, ^38^ or even 20 years after treatment.^15^ In addition, studies have shown white matter damage at 6 months post‐chemotherapy, recovery over 2 years, and then white matter damage again at 3 years after treatment.^14^ However, our study did not include a pretreatment baseline or an acute/early post‐chemotherapy assessment to confirm a potential partial recovery. Nonetheless, previous studies on gray matter density shortly after chemotherapy for breast cancer patients have demonstrated partial recovery of brain structure and function.^39^, ^40^, ^41^, ^42^

We observed a significant positive correlation between the FA value in the left anterior corona radiata and the crystalized composite score in the chemotherapy‐treated group at TP1 but not in the control groups. Prior study has showed that crystalized cognition remains same or slightly higher after middle adulthood in the normal control population.^43^ Additionally, we found no significant correlation from TP1 to TP2, which should not be surprising as the crystalized composite score has been known to be resilient to change.^44^ It was therefore not surprising that no correlation was found in the control groups. Further research with a larger sample size is needed to validate the association between white matter microstructure and cognition in older cancer survivors who have undergone chemotherapy.

In this study, the older long‐term survivors without chemotherapy also showed significantly decreased FA in the right corona radiata, thalamic radiation, and splenium of the corpus callosum. Although the cancer controls in our cohort did not have chemotherapy, 70% of them were treated with hormonal therapy. A recent study showed structural and functional deficits in breast cancer patients treated with hormonal therapy.^45^, ^46^ The effect of hormonal therapy and CRCI deserves further research, especially in older cancer survivors.

There were several limitations to this study. First, this pilot study had a small sample size, and we experienced severe attrition during the 2‐year study period. A larger sample size may detect more regions of alteration in the white matter, which should expand our understanding of neural correlates for CRCI in older long‐term survivors. Second, our study assessed longitudinal changes over a 2‐year interval in long‐term survivors. However, we did not have pretreatment data nor data shortly after chemotherapy to gauge the trajectory of white matter changes from baseline to acute phase after chemotherapy and then through long‐term survivorship. Due to the explanatory nature of this pilot study, we couldn't perform the power analysis to adequately adjust the power of the samples. For the reason that this exploratory pilot study primarily aims to provide any potential white matter changes, we were unable to perform a power analysis for a proper adjustment of sample size. Nevertheless, the insights gained from the exploratory substudy will help to design a larger multi‐central study with suitably adjusted power analysis. Despite these limitations, our study had merits. It was the first longitudinal study of white matter microstructure in older long‐term survivors, which should advance our understanding of CRCI in cancer survivors.

## 5 CONCLUSIONS

We found diminished white matter microstructural integrity in older long‐term breast cancer survivors who received chemotherapy 5–15 years ago. In addition, we also found a significant correlation between white matter and cognitive function, implicating that these white matter alterations may potentially serve as a neural correlate for CRCI in older long‐term survivors.

## AUTHOR CONTRIBUTIONS

**Ebenezer Daniel:** Data curation (equal); formal analysis (equal); software (lead); writing – original draft (lead); writing – review and editing (lead). **Frank Deng:** Data curation (equal); formal analysis (equal); software (supporting); writing – original draft (supporting); writing – review and editing (supporting). **Sunita K. Patel:** Conceptualization (lead); data curation (equal); formal analysis (equal); methodology (lead); writing – review and editing (lead). **Mina S. Sedrak:** Data curation (equal); formal analysis (equal); writing – review and editing (supporting). **Heeyoung Kim:** Data curation (equal); formal analysis (equal); writing – review and editing (supporting). **Marianne Razavi:** Data curation (equal); formal analysis (equal); writing – review and editing (supporting). **Can‐lan Sun:** Data curation (equal); formal analysis (equal); writing – review and editing (equal). **James C. Root:** Data curation (equal); formal analysis (equal); writing – review and editing (lead). **Tim A. Ahles:** Conceptualization (lead); data curation (equal); formal analysis (equal); funding acquisition (lead); methodology (lead); writing – review and editing (lead). **William Dale:** Data curation (equal); formal analysis (equal); funding acquisition (lead); writing – review and editing (supporting). **Bihong T. Chen:** Conceptualization (lead); data curation (lead); formal analysis (lead); funding acquisition (lead); methodology (lead); writing – original draft (lead); writing – review and editing (lead).

## FUNDING INFORMATION

This study was partially funded by National Institutes of Health/National Institute on Aging grants R01 CA172119 (TA), U54 CA137788 (TA), P30 CA008748 (TA), and K24 AG055693‐01 (WD). BTC received funding support from the City of Hope Center for Cancer and Aging.

## CONFLICT OF INTEREST STATEMENT

The authors had no relevant financial or non‐financial interests to disclose.

## ETHICS STATEMENT

All procedures involving human participants were performed in accordance with the ethical standards of the Institutional Review Board of the City of Hope and with the 1964 Helsinki Declaration and its later amendments, as well as with all local, state, and federal regulations. Informed consent was obtained from all participants in the study. The parent study for this neuroimaging substudy has been registered on ClinicalTrials.gov (NCT02122107).

## CONSENT TO PARTICIPATE

Informed consent was obtained from all individual participants included in the study.

## Supporting information


Table S1.
Table S2.
Table S3.

## Data Availability

The datasets generated during the current study are not publicly available due to a lack of relevant public database to deposit the data, but are available from the corresponding author on reasonable request.
