# Supplementary material for: Brain white matter microstructural changes in chemotherapy‐treated older long‐term breast cancer survivors
Source: Cancer Med. 2023 Dec 28;13(1):e6881. doi: 10.1002/cam4.6881 (PMC10807556; doi:10.1002/cam4.6881)
Supplement: Supplementary file 1 — Table S1. Table S2. Table S3. [file CAM4-13-e6881-s001.docx]

**Supplementary Files:**

**Table S1.** Association analysis between the length of time in years after chemotherapy and the changes in white matter tracts.

| **White matter tracts** | Bivariate correlation (p value) | |
| --- | --- | --- |
|  | Pearson correlation  (Parametric correlation) | Spearman’s rho correlation (Nonparametric correlation) |
| Anterior corona radiata Left | 0.83 | 0.80 |
| Body of corpus callosum | 0.66 | 0.53 |
| Genu of corpus callosum | 0.62 | 0.64 |
| External Capsule Left | 0.53 | 0.62 |

**Table S2.** Association analysis between the years since cancer diagnosis and the changes in white matter tracts.

| **White matter tracts** | Pearson correlation  (Parametric correlation) | Spearman’s rho correlation (Nonparametric correlation) |
| --- | --- | --- |
| Anterior corona radiata Left | 0.38 | 0.39 |
| Body of corpus callosum | 0.50 | 0.51 |
| Genu of corpus callosum | 0.92 | 0.91 |
| External Capsule Left | 0.42 | 0.39 |

**Table S3.** Summary of neuropsychological testing data obtained with NIH toolbox cognition battery.

| **NIH toolbox score** | **C+ group** | | | **C- group** | | | **HC group** | | |
| --- | --- | --- | --- | --- | --- | --- | --- | --- | --- |
|  | **Mean** | | **df** | **Mean** | | **df** | **Mean** | | **df** |
|  | *TP1* | *TP2* | *p* | *TP1* | *TP2* | *p* | *TP1* | *TP2* | *p* |
| Total composite score | 58.44 | 54.47 | **0.01** | 60.54 | 58.97 | 0.29 | 60.97 | 59.87 | 0.42 |
| Fluid composite score | 53.50 | 49.06 | **0.03** | 51.97 | 50.30 | 0.40 | 53.42 | 51.31 | 0.25 |
| Crystallized composite score | 60.65 | 58.41 | 0.057 | 65.74 | 64.98 | 0.52 | 64.99 | 64.99 | 0.99 |
| Picture vocabulary score | 59.88 | 56.13 | 0.04 | 64.14 | 64.35 | 0.91 | 64.88 | 65.09 | 0.89 |
| Picture sequence memory score | 59.10 | 54.11 | **0.05** | 52.95 | 51.14 | 0.49 | 54.78 | 53.55 | 0.61 |
| Pattern comparison process. speed score | 44.82 | 41.25 | 0.29 | 45.04 | 41.46 | 0.30 | 42.68 | 39.97 | 0.39 |
| Oral reading recognition score | 59.37 | 58.96 | 0.73 | 64.43 | 62.34 | 0.11 | 62.14 | 61.87 | 0.81 |
| List sorting working memory score | 53.33 | 52.64 | 0.72 | 51.39 | 52.68 | 0.52 | 55.35 | 54.69 | 0.72 |
| Flanker inhibitory control score | 50.16 | 47.58 | 0.28 | 49.82 | 50.10 | 0.90 | 52.55 | 48.44 | 0.07 |
| Dimensional change care sort score | 55.05 | 53.13 | 0.42 | 58.02 | 55.71 | 0.35 | 56.91 | 55.21 | 0.45 |

**Abbreviations:** C+: Chemotherapy group; C-: No-chemotherapy group; HC: Healthy control group; TP1: Time point 1; TP2: Time point 2 and df: difference between TP1 and TP2. The p value is given for the longitudinal changes between TP1 and TP2. A significant level of threshold is p<0.05.
